# MBind: A Web-Based Platform for Metalloprotein and Nonmetalloprotein Docking with Optional ML Docking Integration

**DOI:** 10.3390/molecules31152703

**Published:** 2026-08-03

**Authors:** Harrish Ganesh, Sahith Mada, Suhani Aryal, Ronan Zwa, Karen Lainez Diaz, Ishaan Patel, Sivanesan Dakshanamurthy

**Affiliations:** 1 VCU Life Sciences, Virginia Commonwealth University, Richmond, VA 23284, USA; 2 College of Computer, Mathematical, and Natural Sciences, University of Maryland, College Park, MD 20742, USA; 3 School of Public Health, University of Maryland, College Park, MD 20742, USA; 4 Department of Molecular Biology, Cell Biology, and Biochemistry, Brown University, Providence, RI 02912, USA; 5 School of Neuroscience, College of Science, Virginia Polytechnic Institute and State University, Blacksburg, VA 24061, USA; 6 Center for the Study of Human Health and Department of Economics, Emory College of Arts and Sciences, Emory University, Atlanta, GA 30322, USA; 7 Lombardi Comprehensive Cancer Center, Georgetown University Medical Center, Washington, DC 20007, USA; 8 Innscite AI LLC, Herndon, VA 20170, USA

**Keywords:** MBind, metalloproteins, metal docking, AutoDock Vina, AutoDock4, Streamlit interface, binding free energy analysis

## Abstract

Metalloprotein docking is difficult because it requires precise preparation of the grid box, charge assignment, and metal coordination geometry. Many current graphical user interfaces (GUIs) do not support these steps well. Here, we present MBind v2026, a web-based GUI that combines AutoDock Vina, AutoGrid4 and AutoDock4 into a single workflow for standard docking and metalloprotein docking. The interface also has the optional machine learning (ML) docking program GNINA v1.0. The main validated metalloprotein workflow in MBind is based on AutoDock4Zn parameterization for zinc, while preliminary workflows for magnesium, iron, and copper are also included. MBind was benchmarked on eight zinc metalloproteins and eight nonmetalloprotein targets. Its performance was compared with ML pose prediction methods (GNINA v1.0, EquiBind v2026, TankBind v2026, and GAABind v2026), cofolding models (Boltz-2 v2026, and AlphaFold 3 v2026), ML affinity prediction tools (StructureNet v2026, GNNSeq v2026, and PLAIG v2026), and non-ML docking tools (SwissDock v2026, CB-Dock2 v2026, Webina v2026, 1-ClickDock v2026, and MolModa v1.01). Pose accuracy was measured by symmetry-aware ligand RMSD using DockRMSD v1.1 under redocking conditions with co-crystal-defined binding sites. Binding energy trends were evaluated by comparing docking scores with IC50-derived ΔG values using mean absolute error. On the zinc metalloprotein benchmark set, MBind produced a mean RMSD of 0.49 Å. On the nonmetalloprotein set, the mean RMSD was 0.62 Å. These results show that MBind can execute zinc metalloprotein and nonmetalloprotein docking workflows through a web-based interface, while the preliminary Mg, Fe, and Cu workflows require additional validation. The MBind GUI web-based platform is publicly available.

## 1. Introduction

Molecular docking is used to predict ligand-receptor binding interactions [1,2,3]. The metalloprotein docking process remains challenging due to metal coordination, charge assignment, and local electrostatics, which are required for accurate results [4,5,6]. Additionally, molecules involved in metalloprotein docking primarily interact with proteins using noncovalent interactions [7,8,9]. Standard docking pipelines were largely developed for proteins without catalytic metal ions and may underperform when a metal is present in the binding site [10,11,12]. In these workflows, incorrect atom typing or metal parameterization can produce inaccurate coordination distances and ligand poses [13,14,15,16,17]. Several docking packages, such as GOLD and the FITTED Suite, include metal-aware scoring that incorporates coordination geometry and related constraints [18,19]. AutoDock4Zn was developed to improve zinc metalloprotein docking by adding atom types and forcefield parameters specific to zinc [20]. However, these workflows are typically used via command line interfaces and require manual atom typing and charge assignment for metals [21]. Small errors in these steps can affect docking outcomes, because map generation and docking execution are handled by separate software packages [22,23]. This increases the likelihood of inconsistent settings between programs and reduces reproducibility [24]. Many AutoDock GUIs and web servers focus on conventional protein docking, which requires users to write external scripts to prepare specific metal ions. Additionally, many platforms do not directly support AutoDock4 (AD4), AutoDock Vina, and ML docking output formats, which forces manual file management and conversion. These limitations show that current tools do not provide a single workflow that automates metal-specific preprocessing, consistent parameter handling, and output organization among AD4, AutoDock Vina, and ML-based docking methods [18,19].

Here, we present MBind (v2026, https://mbindgui.streamlit.app/) not simply as a GUI. It is a unified docking workflow that automates preprocessing of metalloproteins from PDBQT structure files and integrates AutoDock4, AutoDock Vina, and GNINA v1.0 within a single reproducible platform for both metalloproteins and nonmetalloproteins. MBind primarily supports AutoDock4Zn-style zinc preparation through zinc typing and grid map generation using AD4Zn.dat. It also provides basic support for copper, iron, and magnesium metalloproteins. MBind runs AD4 and AutoDock Vina within the same workflow with automatic output organization. Additionally, MBind integrates GNINA as an optional ML-assisted docking method within the same interface. Together, MBind becomes an integration- and usability-focused platform that brings metal-specific setup, consistent parameter handling, and output organization into a single workflow for metalloprotein and nonmetalloprotein protein docking.

To evaluate this integrated workflow, MBind was compared against established ML and conventional docking methods. We benchmarked eight zinc metalloproteins and eight nonmetalloproteins and compared its performance with nine ML methods. Pose prediction ML methods included GNINA, EquiBind (v2026, https://github.com/HannesStark/EquiBind), TankBind (v2026, https://github.com/luwei0917/TankBind) and GAABind (v2026, https://github.com/Mercuryhs/GAABind) [25,26,27,28]. ML cofolding models involved Boltz-2 (v2026, https://github.com/jwohlwend/boltz) and AlphaFold 3 (v2026, https://alphafoldserver.com/welcome) [29,30], and ML affinity prediction tools incorporated StructureNet (v2026, https://github.com/sivaGU/StructureNet), GNNSeq (v2026, https://github.com/sivaGU/GNNSeq), and PLAIG (v2026, https://plaig-demo.streamlit.app/) [31,32,33]. Non-ML docking methods were also compared against Mbind, SwissDock (v2026, https://www.swissdock.ch/), CB-Dock2 (v2026, http://cao.labshare.cn:11080/cb-dock2/php/blinddock.php), Webina (v2026, https://durrantlab.pitt.edu/webina/), 1-ClickDock (v2026, https://mcule.com/apps/1-click-docking/) and MolModa v1.01 [34,35,36,37,38]. MBind was benchmarked against these ML and non-ML docking tools under a fixed redocking protocol. We report pose accuracy under redocking conditions using symmetry-aware RMSD and compared model scores to IC50-derived ΔG estimates as an approximate analysis. MBind showed lower mean RMSD for metalloproteins, while its scores showed agreement with the IC50-derived ΔG estimates similar to that of several compared methods. MBind’s unified workflow supports docking execution and pose evaluation for PDBQT metalloprotein and nonmetalloprotein structure inputs, while supporting the development and testing of metalloprotein docking parameterizations.

## 2. Results and Discussion

### 2.1. Overview of MBind Capabilities

**Figure 1:**
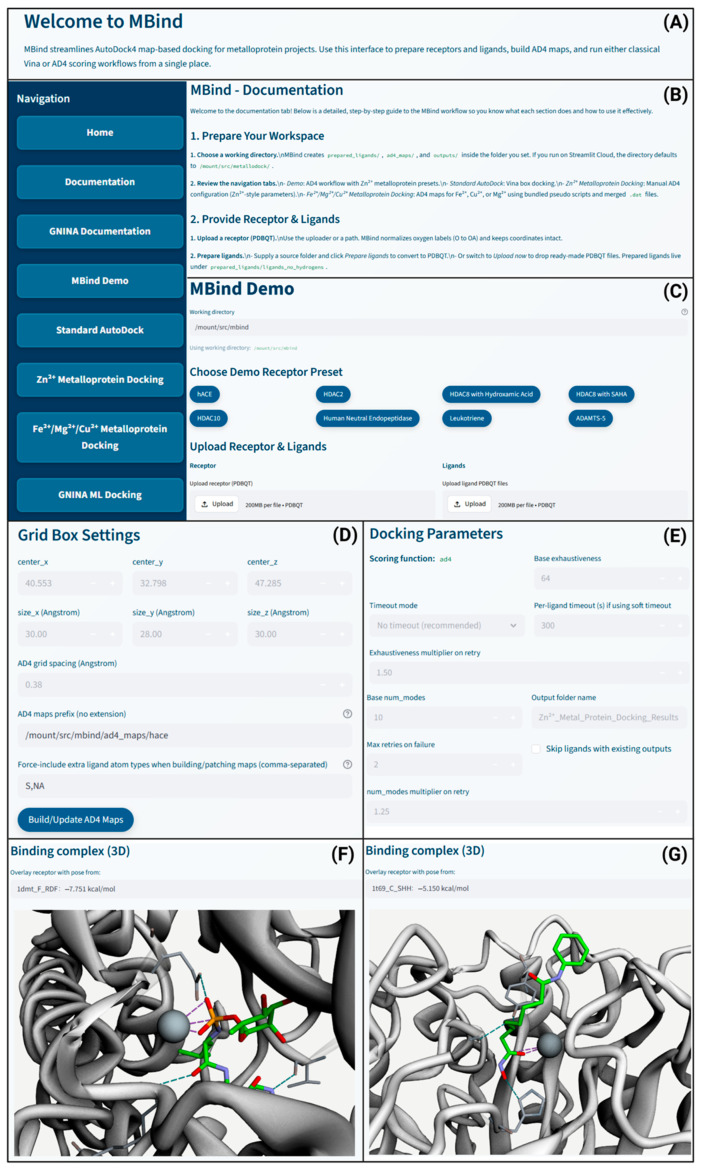
MBind graphical user interface for nonmetalloprotein and metalloprotein docking with electrostatics output. MBind is a Streamlit-based web application that combines AutoDock Vina and AD4, so both conventional and Zn metalloprotein (and preliminarily Cu, Fe, and Mg) docking runs are easy to set up and can be configured and executed in the GUI. (**A**) Landing page summarizing the purpose of MBind. (**B**) Navigation sidebar used to move between the Home, Documentation, Demo, Standard AutoDock, and Metalloprotein Docking workflows. (**C**) Demo workspace where users select a receptor preset, specify the working directory, and upload receptor and ligand PDBQT files for docking. (**D**) Gridbox and AD4 map controls, which include center and size coordinates, grid spacing, map prefix, and options to create AD4 maps while forcing the inclusion of specified atom types for metalloprotein projects. (**E**) Docking parameter panel, where users choose the scoring function (Vina or AD4), set base exhaustiveness and per-ligand timeout, apply an exhaustiveness multiplier on retry, and launch docking jobs. (**F**) Protein visualization feature showing metalloprotein docking results of Human Neutral Endopeptidase. (**G**) Protein visualization feature showing metalloprotein docking results of HDAC8 with SAHA. Dashed lines indicate distance-based visualization guides: teal for contacts involving polar AD4 atom types, green for carbon–carbon contacts involving aromatic residues, slate for other close ligand–residue contacts, and purple for metal–ligand contacts.

The MBind interface is organized into structured tabs that guide the user through each step of the docking process. Users begin by uploading receptor files in PDBQT format. Grid box centers and dimensions can then be defined directly within the GUI. The metalloprotein workflow includes automatic AD4 map generation and supports metal coordinating atom types needed for zinc docking. The grid generation code creates electrostatic maps using AutoGrid commands within the interface. Therefore, users do not need to manually create AD4 parameter files or provide a zinc force field file. A visualization of the GUI and description is shown below (Figure 1). MBind features navigation tools and documentation directly on the platform (Figure 1A,B). MBind also supports both single- and multi-ligand workflows. Users can upload multiple ligands, and the platform processes them sequentially using a controlled execution loop (Figure 1C). Each ligand is docked using user-defined Vina parameters such as exhaustiveness, number of output poses, and timeout duration, with settings available for adjusting the grid box (Figure 1D,E). Visualization of docking outputs is also supported (Figure 1F,G). Detailed instructions on navigating the website and using MBind are available directly on the site at https://mbindgui.streamlit.app/ (accessed on 29 July 2026).

### 2.2. MBind Usability Case Studies

#### 2.2.1. Case Study 1: MBind Zinc Metalloprotein and Nonmetalloprotein Docking

MBind was tested by a user with little computational experience to measure the usability of the tool. The user was able to complete docking through the GUI in a simple sequence of steps using pre-prepared receptor and ligand PDBQT files. After uploading a receptor, ligand, and starting the run, results were obtained within minutes. For zinc metalloproteins, the platform handled the required setup steps internally. This included AutoDock4Zn preprocessing and run preparation, so the user did not need to manually create parameter files. The same user then performed a nonmetalloprotein docking run in the same interface using the Vina workflow, with grid setup, run execution, and output retrieval all managed in the GUI. This shows that MBind can be used consistently for metalloprotein and nonmetalloprotein cases without switching tools or using command line prompts.

#### 2.2.2. Case Study 2: MBind Integration of ML Docking Through the GUI

MBind was used by a user seeking to apply ML-based docking methods without command line interaction. The user selected the GNINA workflow directly from the GUI and executed ML pose and energy prediction using the same prepared ligand and receptor inputs used for nonmetalloprotein docking. MBind handled input formatting and output organization automatically to allow the user to obtain ML docking results within the same interface. This case study shows MBind’s ability to integrate ML docking methods into a unified workflow.

#### 2.2.3. Case Study 3: MBind Classroom Use

MBind was used in a teaching setting to represent metalloprotein docking concepts. Students were able to complete zinc docking runs within minutes using the web-based interface. This allowed instruction to focus on the understanding of binding poses rather than software setup. This shows MBind’s applicability for educational and exploratory applications where traditional workflows may be challenging due to the unfamiliarity for many individuals.

#### 2.2.4. Case Study 4: Development of Preliminary Mg^2+^, Cu^2+^, and Fe^2+^ Docking Parameters

MBind was used to explore the development of preliminary docking parameters for Mg^2+^, Fe^2+^, and Cu^2+^ metalloproteins. Established AutoDock parameterizations similar to AutoDock4Zn are not currently available for many of these metal complexes. To address this gap, prototype force fields were constructed by adapting existing AutoDock force fields. Interaction parameters were then modified to approximate metal-specific coordination behavior. These parameters were implemented within the MBind environment so that users could perform exploratory docking runs on Mg^2+^, Fe^2+^, and Cu^2+^ containing proteins through the same GUI-based workflow used for other docking methods.

The current implementations should be considered experimental and not fully validated. The coordination chemistry of Mg^2+^, Fe^2+^, and Cu^2+^ differs significantly from zinc. As a result, the present parameter sets primarily serve to show that MBind can assist in the development and testing of new metalloprotein force fields within the same interface. Future work will focus on improving parameter accuracy through calibration with experimental structural and binding affinity data.

### 2.3. Performance Validation of MBind

MBind was benchmarked with both metalloproteins and nonmetalloprotein targets. This was done to assess native pose recovery under redocking conditions. A separate exploratory analysis compared model scores with BFE values estimated from reported inhibitory activity. Known co-crystal ligands were redocked into each protein’s binding site (Table 1). Pose accuracy was measured using RMSD calculated with DockRMSD. This tool matches corresponding ligand atoms while factoring molecular symmetry, and predicts the minimum RMSD relative to the native pose [39]. During benchmark docking, the automated retry function was disabled by selecting the no timeout mode to allow each docking job to run to completion without time limits. As a result, no docking jobs required retries, and all benchmarking results are single-pass docking runs.

Reported IC_50_ values were transformed at 298 K using Equation (1) to generate approximate reference ΔG values. This was necessary due to the lack of experimental ΔG, K_i_, K_d_, or K_m_ values for certain proteins. The use of IC_50_ thus limits assessment of absolute BFE prediction accuracy. Analysis of rank agreement was used to increase the validity of comparisons between methods. Experimental BFEs were used as reference values for MBind predicted docking scores. For MBind, predicted docking scores corresponded to AutoDock Vina or AD4 scoring outputs depending on the selected workflow. These scores were treated as relative energy estimates and compared directly to IC50-converted ΔG values without additional calibration. Additionally, BFE error was defined as the absolute difference between the predicted ΔG and the closest corresponding experimental ΔG value. When experimental IC_50_ values were reported as intervals rather than single values, BFE error was calculated as the absolute deviation from the nearest ΔG value calculated from the reported IC_50_ range. This rule was applied consistently for all docking methods and benchmark targets. For the metalloprotein benchmark set, IC_50_ intervals were reported for HDAC2, hACE, Human Neutral Endopeptidase, and LTA4H. For the nonmetalloprotein benchmark set, IC_50_ intervals were reported for AR, Hsp90 with geldanamycin, and MDM2. Specific ligands with their respective experimental and predicted binding energies, as well as RMSD differences between predicted and native binding poses, are reported in Table S3a,b.

(1) ΔG=RT ln(K)

**Figure 2:**
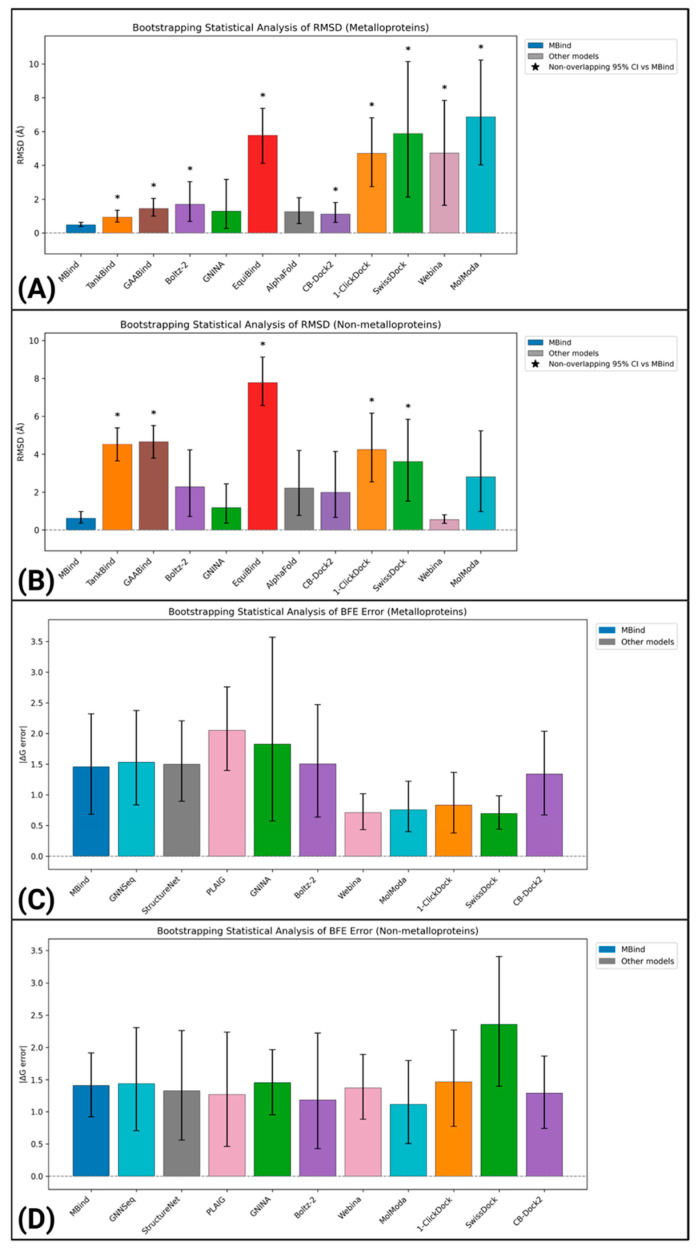
Bootstrap statistical analysis of docking performance between methods. (**A**,**B**) Ligand pose accuracy measured by mean ligand RMSD (Å) relative to the native co-crystal conformation. (**C**,**D**) BFE prediction error measured as absolute error relative to experimental IC50-derived ΔG values. Bars represent mean values computed from 5000 bootstrap resamples, and error bars represent 95% confidence intervals. Stars represent models whose confidence intervals do not overlap with MBind. These markers indicate interval separation within the observed benchmark and do not represent formal significance testing. (**A**,**C**) Metalloprotein benchmark set. (**B**,**D**) Nonmetalloprotein benchmark set.

Uncertainty in mean RMSD and BFE error was calculated using nonparametric bootstrap resampling since each benchmark set consisted of a limited number of targets (n = 8 per protein class). For each docking method and benchmark set, 5000 bootstrap resamples were created by sampling targets with replacement, and 95% confidence intervals were calculated for the mean RMSD and BFE error. These intervals describe uncertainty within the observed eight-target samples. They do not overcome the limited sample size or estimate population-level variance. Therefore, they were interpreted descriptively and do not signify statistical significance. Raw confidence interval data and bootstrapping analysis outputs are provided in the supplementary materials (Table S4). Mean ligand RMSD values for the metalloprotein and nonmetalloprotein benchmarks are visualized separately (Figure 2A,B). BFE prediction error relative to experimental IC50-derived ΔG values is also visualized separately for the metalloprotein and nonmetalloprotein benchmarks (Figure 2C,D).

#### 2.3.1. Benchmarking of MBind in Comparison with ML Models

##### Benchmarking of Ligand Pose Accuracy Through RMSD

**Figure 3:**
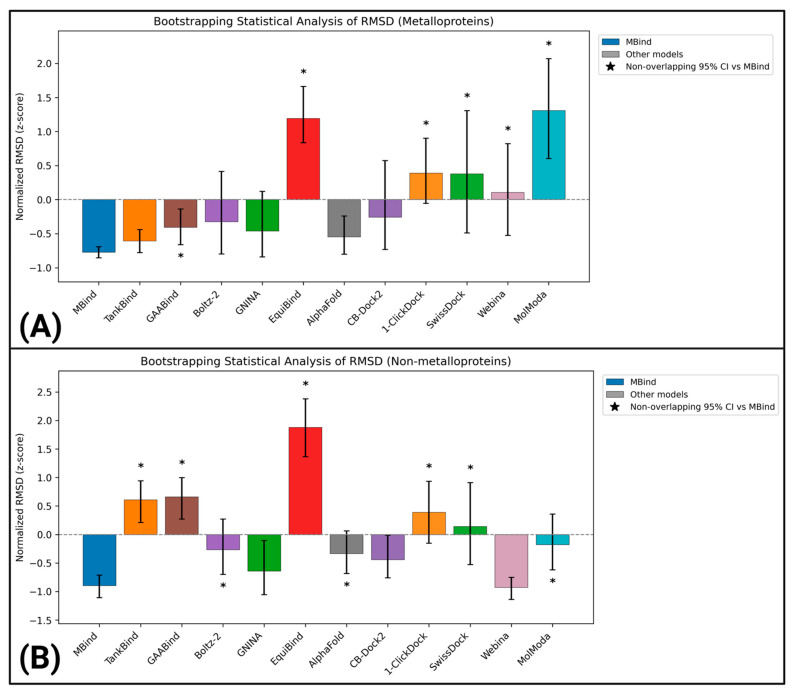
Bootstrap comparison of ligand pose accuracy for docking methods. Bars show standardized mean ligand RMSD (z-scored Å) relative to the native co-crystal conformation using 5000 nonparametric bootstrap resamples. (**A**) Metalloprotein benchmark set. (**B**) Nonmetalloprotein benchmark set.

**Figure 4:**
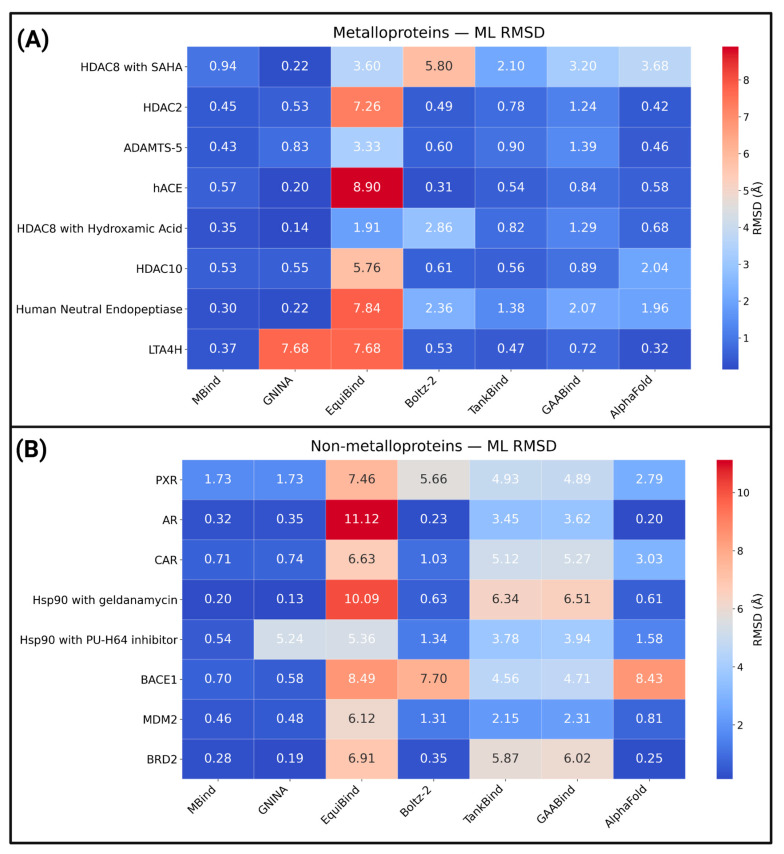
Benchmarking performance for binding pose prediction of ML models compared to MBind. Pose accuracy is reported as ligand RMSD (Å) relative to the native co-crystal conformation for MBind and ML-based docking models for benchmark targets. (**A**) Metalloprotein benchmark set. (**B**) Nonmetalloprotein benchmark set.

MBind produced noticeably lower RMSD values (<0.7 Å) on zinc metalloprotein targets when compared to most ML models used for pose prediction (Figure 3A). MBind showed a lower observed mean RMSD than most ML pose-prediction baselines (Figure 3A). Pose accuracy for all RMSD comparisons was measured using the rank 1 pose selected by each method. All pose accuracy benchmarks were performed under redocking conditions using the co-crystal-defined binding sites. Among the ML methods, TankBind performed the closest to MBind. However, EquiBind showed the greatest deviation, while performance for GAABind, Boltz-2, and GNINA fell in between (Figure 4A). MBind’s average RMSD of 0.492 ± 0.202 Å was notably lower than the mean of 2.074 ± 2.396 Å seen collectively among the six ML models, which shows lower observed pose error within this redocking benchmark. AlphaFold 3 showed generally low RMSD values for several metalloprotein targets, but its performance varied among the datasets and remained less consistent than MBind (Figure 4A). These results show lower observed mean pose error for MBind under the tested redocking conditions in relation to the metalloprotein dataset (Figure 3A). ESMFold2 was excluded because it lacks the metal-specific potentials needed for accurate metalloprotein docking.

GNINA performed best in pose prediction among the ML models tested on the nonmetalloprotein benchmark set. Boltz-2 and AlphaFold showed moderate performance, while TankBind, GAABind, and EquiBind showed reduced accuracy (Figure 4B). Among the six ML models benchmarked, nonmetalloprotein pose prediction showed a mean RMSD of 3.773 Å with a standard deviation of 2.959 Å. MBind was associated with a lower observed mean RMSD in the nonmetalloprotein set, with an average RMSD of 0.619 Å (Figure 4B). Within the nonmetalloprotein subset, MBind had a lower mean RMSD relative to TankBind, GAABind, 1-ClickDock, and SwissDock (Figure 3B). These benchmarking results show lower observed pose error for MBind than several methods under the tested redocking protocol. However, MBind’s confidence interval overlapped with the confidence intervals of Boltz-2, GNINA, CB-Dock2, Webina, and MolModa (Figure 3B). These results show that performance in the nonmetalloprotein set is less consistent compared to the metalloprotein benchmark. Here, several methods showed comparable performance to MBind. This is expected, as MBind uses standard AutoDock Vina-based workflows for nonmetalloprotein complexes. In these cases, improvements from metal-aware parameterization are not a factor. MBind showed lower observed ligand RMSD under redocking conditions for metalloproteins and most nonmetalloprotein complexes compared to ML models.

##### Benchmarking of Binding Energy Prediction Accuracy

**Figure 5:**
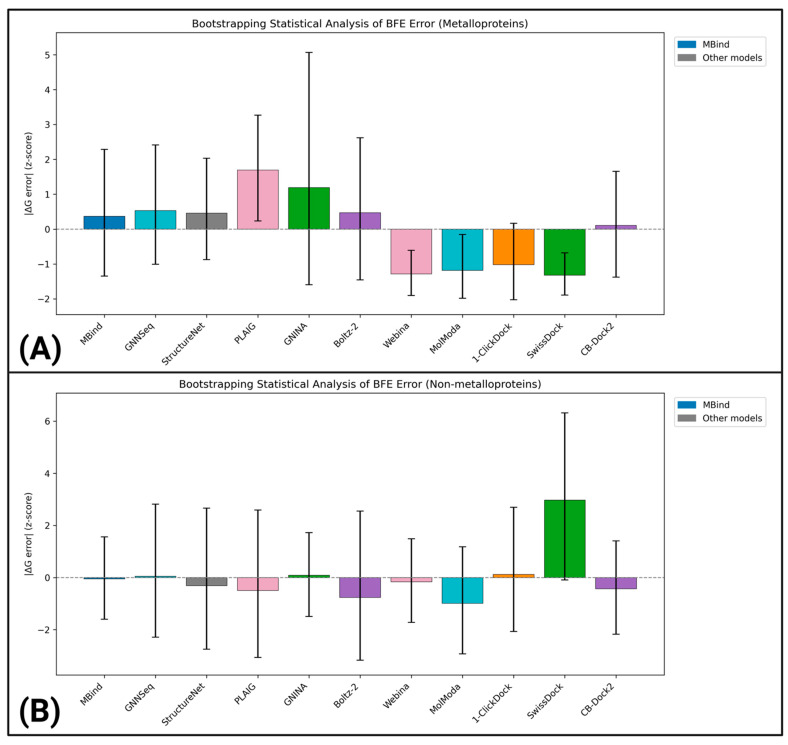
Bootstrap comparison of BFE prediction error between docking methods. Bars show standardized mean absolute error (z-scored |ΔG error|) relative to experimental IC_50_-derived ΔG values from 5000 nonparametric bootstrap resamples. (**A**) Metalloprotein benchmark set. (**B**) nonmetalloprotein benchmark set.

MBind showed similar mean absolute error in ΔG compared with the ML methods on the metalloprotein benchmark (Figure 5A). The absolute difference between model-reported docking or affinity scores and ΔG values was calculated from experimental IC_50_ values (Figure 5A). Vina-based scores and ML model outputs were seen as ranking features rather than thermodynamic free energies.

**Figure 6:**
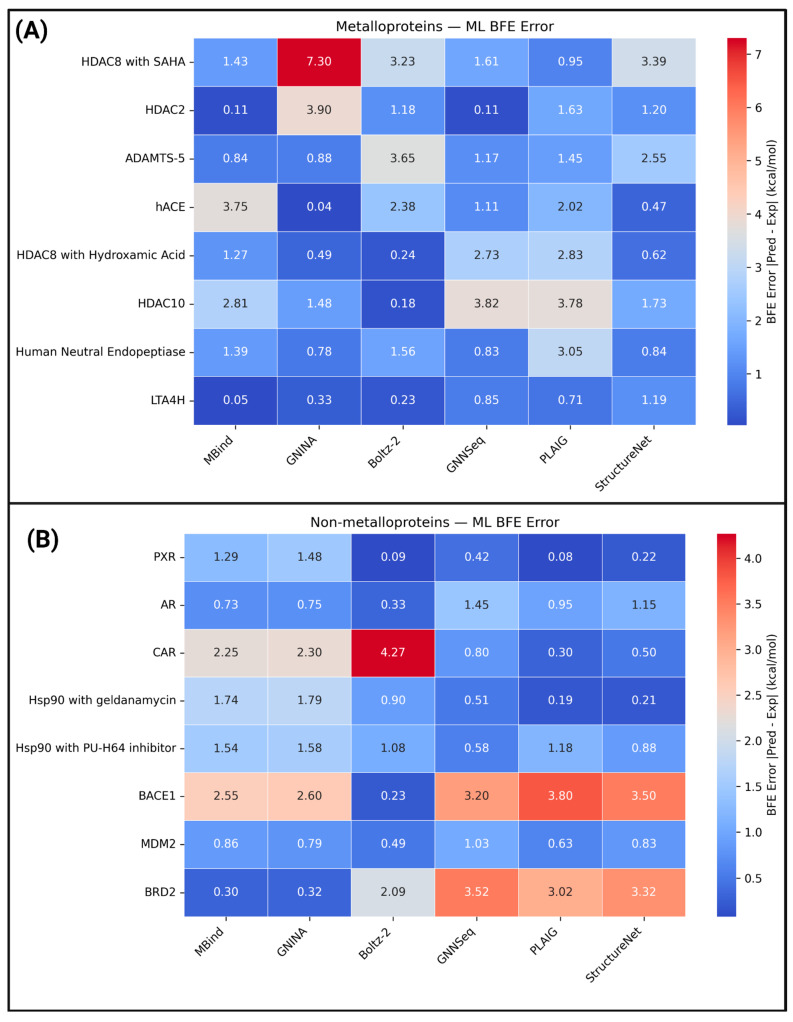
Benchmarking performance for BFE prediction of ML models compared to MBind. BFE error relative to experimental ΔG for MBind and ML-based docking models for benchmark targets. (**A**) Metalloprotein benchmark set. (**B**) Non-metalloprotein benchmark set.

**Figure 7:**
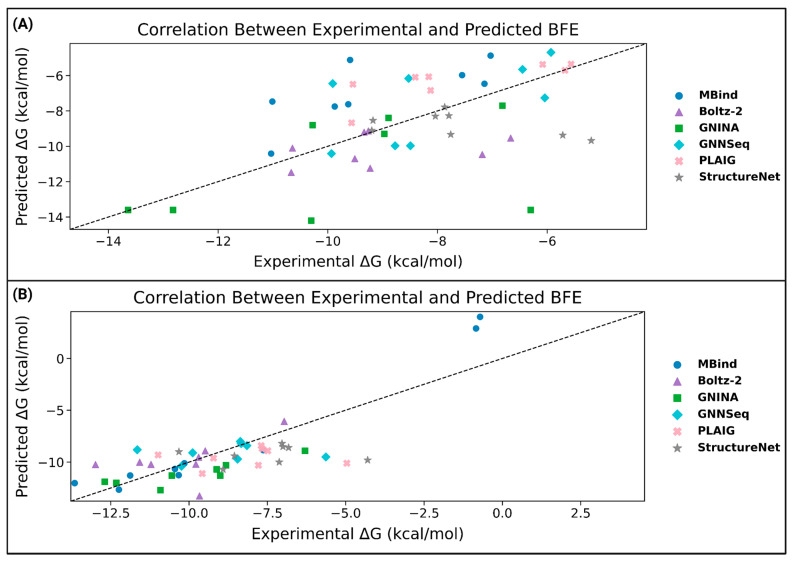
The benchmarking performance for BFE prediction of ML docking tools compared to MBind. Correlation between experimental and predicted BFE (ΔG, kcal/mol) is shown for MBind and ML docking platforms for benchmark targets, with the dashed line showing ideal agreement (y = x). (**A**) Metalloprotein benchmark set. (**B**) Nonmetalloprotein benchmark set.

Among ML platforms, the BFE prediction error was generally moderate among most targets (Figure 6A). GNINA, GNNSeq, PLAIG, and StructureNet showed similar overall behavior, with consistent error among most complexes but increased deviations for specific targets such as HDAC8 with SAHA. Boltz-2 showed lower error for select targets, including HDAC10 and LTA4H, but higher error for HDAC8 with SAHA and ADAMTS-5. StructureNet also followed this trend, with larger deviations observed for hACE and HDAC8 with SAHA (Figure 6A). Bootstrap confidence intervals for mean BFE error were consistent among ML methods, which show similar average performance under resampling despite differences in mean error (Figure 5A). The corresponding experimental and predicted ΔG correlation shows that MBind performs similarly to the other ML methods for the metalloprotein benchmark (Figure 7A). Overall, MBind performed comparably to the ML models tested, while maintaining a slightly lower average BFE error.

Additionally, in the nonmetalloprotein benchmark set, MBind showed similar BFE prediction accuracy relative to experimental ΔG values compared to other ML models (Figure 7B). GNINA produced similar results to MBind for several nonmetalloprotein targets, which can be expected since both platforms rely on AutoDock Vina scoring (Figure 7B). Performance among the remaining platforms was more varied. Boltz-2 showed high variability, with high error for CAR but lower error for other targets (Figure 6B). Moderate performance was produced by GNNSeq, PLAIG, and StructureNet overall, though these models showed higher error for BACE1 and BRD2 (Figure 6B). Bootstrap confidence intervals overlapped for ML methods, which shows comparable mean BFE prediction performance under resampling (Figure 5B). When compared to the ML models tested, MBind had similar BFE predictions to conventional docking (Figure 10B).

#### 2.3.2. Benchmarking of MBind with Non-ML Tools

##### Benchmarking of Ligand Pose Accuracy Through RMSD

**Figure 8:**
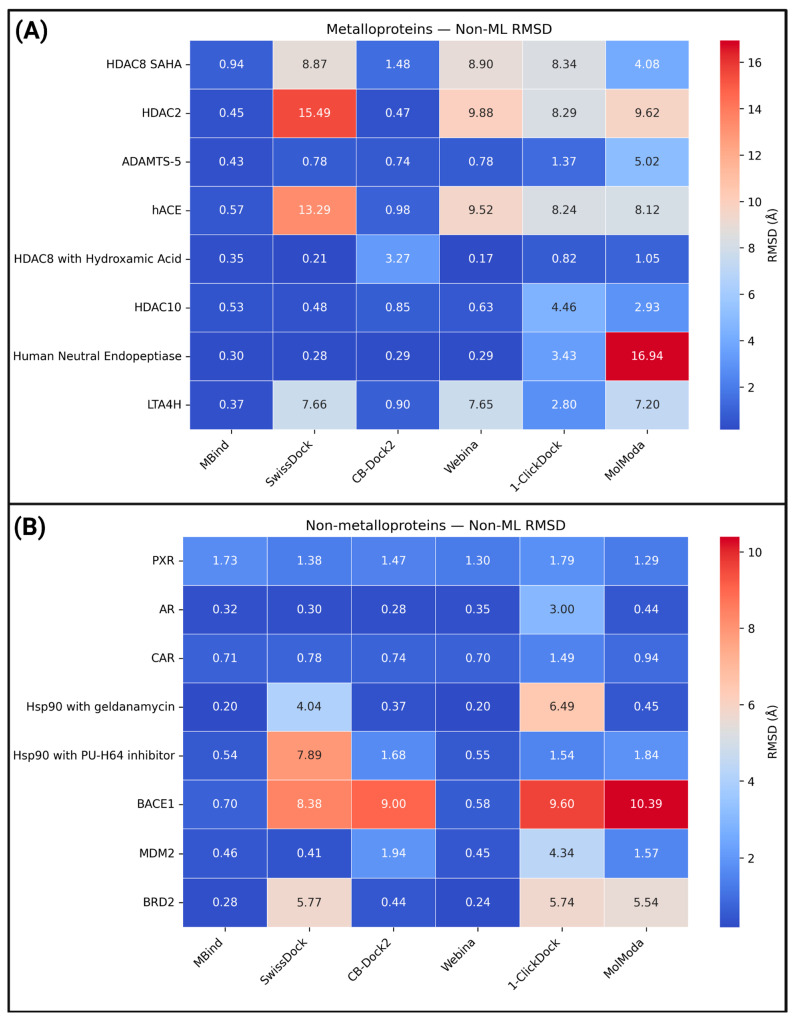
Benchmarking performance for binding pose prediction of non-ML docking tools compared to MBind. Pose accuracy is reported as ligand RMSD (Å) relative to the native co-crystal conformation for MBind and non-ML docking platforms for benchmark targets. (**A**) Metalloprotein benchmark set. (**B**) Nonmetalloprotein benchmark set.

Alongside the eight ML platforms, MBind was also benchmarked against five non-ML docking tools. Among the eight metalloproteins tested, MBind produced consistently lower RMSD values compared to the tested non-ML platforms (Figure 3A). Among the non-ML tools benchmarked, CB-Dock2 performed the closest to MBind, while MolModa showed the greatest deviation. SwissDock, Webina, and 1-ClickDock performances fell in between (Figure 8A). Collectively, pose prediction accuracy for the five non-ML platforms showed a mean of 4.664 Å with a standard deviation of 2.175 Å. In contrast, the average RMSD for MBind was 0.492 Å for metalloproteins, with a standard deviation of 0.202 Å (Figure 8A). Thus, based on RMSD-based pose accuracy, MBind showed lower observed pose error within this metalloprotein redocking benchmark, where integrated preparation and metal-aware docking are needed.

The non-ML tools showed a more varied performance relative to MBind within the nonmetalloprotein benchmark set. Webina and CB-Dock2 performed most comparably to MBind. In contrast, SwissDock and 1-ClickDock showed higher error, while MolModa’s performance was in between these models (Figure 8B). The non-ML tools showed a mean RMSD of 2.643 Å with a standard deviation of 2.995 Å, while MBind showed lower and more consistent performance (0.619 ± 0.488 Å). MBind showed a similar mean RMSD to the strongest non-ML tools, such as Webina and CB-Dock2, where confidence intervals overlap (Figure 3B). On the other hand, MBind had a lower observed mean RMSD than higher-error non-ML tools, where intervals do not overlap, such as SwissDock and 1-ClickDock (Figure 3B). These results suggest that MBind remains comparable to many conventional docking programs for nonmetalloproteins. However, MBind’s primary advantage is its metal-aware docking preparation.

##### Benchmarking of Binding Free Energy Prediction Accuracy

**Figure 9:**
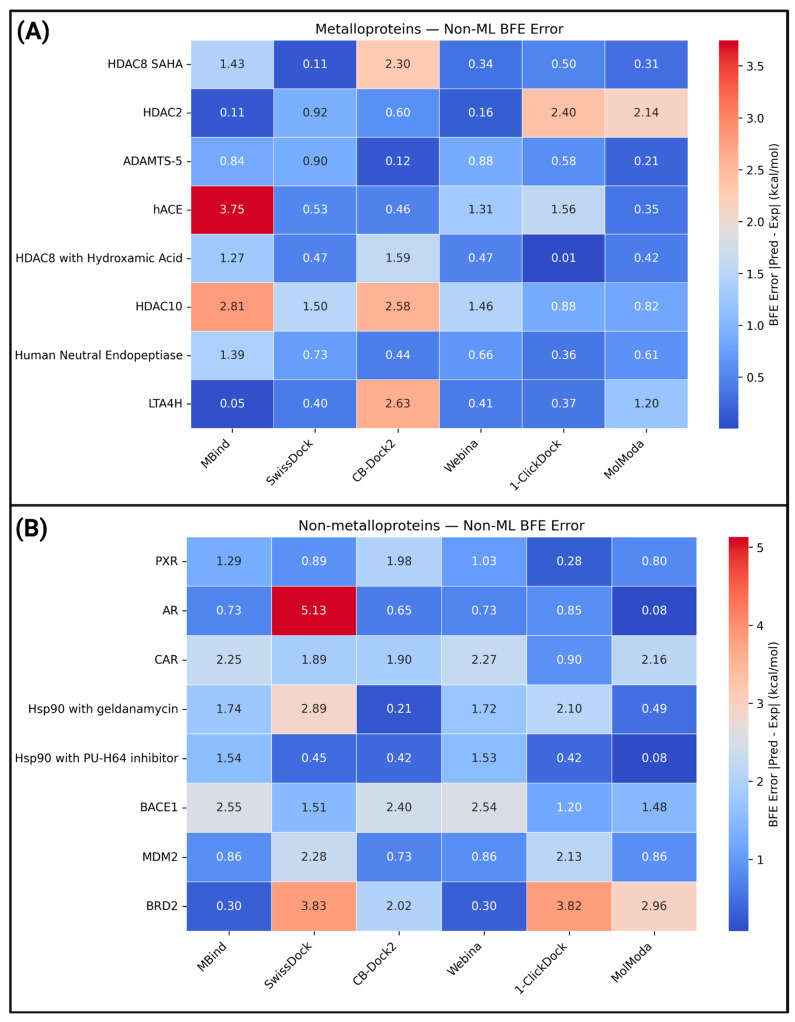
Benchmarking performance for BFE prediction of non-ML models compared to MBind. BFE error relative to experimental ΔG is shown for MBind and non-ML docking models for benchmark targets. (**A**) Metalloprotein benchmark set. (**B**) Nonmetalloprotein benchmark set.

**Figure 10:**
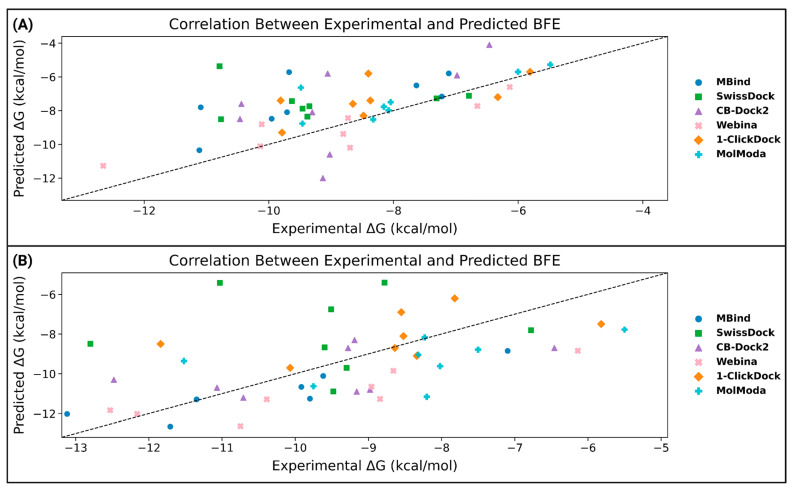
Benchmarking performance for BFE prediction of non-ML docking tools compared to MBind. Correlation between experimental and predicted BFE (ΔG, kcal/mol) is shown for MBind and non-ML docking platforms for benchmark targets, with the dashed line showing ideal agreement (y = x). (**A**) Metalloprotein benchmark set. (**B**) Nonmetalloprotein benchmark set.

MBind showed competitive BFE prediction accuracy for the metalloprotein benchmark set relative to the non-ML docking tools, even without achieving the lowest mean error in all cases. Among the non-ML tools tested, Webina and SwissDock produced slightly lower average error for metalloproteins, but the overall performance remained close for top methods (Figure 9A). SwissDock showed high error for HDAC2, but it produced lower error for targets such as ADAMTS-5 and HDAC8 with Hydroxamic Acid (Figure 9A). Webina also produced low error for several metalloproteins, which include HDAC2 and ADAMTS-5 (Figure 9A). CB-Dock2 showed higher variability due to increased error for HDAC8 with SAHA and LTA4H (Figure 9A). 1-ClickDock showed higher error for HDAC8 with Hydroxamic Acid and HDAC8 with SAHA (Figure 9A). MolModa showed moderate error overall, but it produced higher error for HDAC2. Similar average performance was observed among non-ML methods under resampling (Figure 5A). Thus, MBind shows relatively similar performance for BFE prediction compared to other non-ML models in the metalloprotein dataset. The comparison of experimental and predicted ΔG shows a similar distribution around the ideal agreement line among MBind and the non-ML tools. This indicates that BFE prediction performance is comparable to the other tested methods for metalloproteins (Figure 10A).

Additionally, MBind showed a similar average BFE error relative to experimental ΔG values compared to other non-ML models for the nonmetalloprotein benchmark set (Figure 10B). While CB-Dock2 produced the lowest average error for nonmetalloproteins, MBind and other methods showed comparable performance overall, including similar mean BFE prediction under resampling (Figure 5B). Among methods, variability in BFE prediction was target-dependent. SwissDock showed higher variability, particularly for AR and BRD2, while 1-ClickDock showed increased error for select complexes such as BRD2 and Hsp90 with geldanamycin. MolModa produced moderate error among most nonmetalloprotein targets but did not show clear improvement over CB-Dock2 or Webina (Figure 9B). These results show that MBind achieves BFE prediction performance comparable to existing non-ML docking tools for nonmetalloprotein complexes, with no consistent advantage or disadvantage among targets.

##### Rank Agreement and Relative Energy Interpretation of Docking Scores

Spearman rank correlation was used to calculate rank agreement between predicted docking scores and IC50-derived activity estimates. Mean absolute error was also calculated to measure numerical deviation. When IC_50_ values were reported as ranges, the geometric midpoint of each range was used as a fixed reference value. The same activity reference was used for all methods included in the benchmark. Using IC_50_ to calculate binding free energy estimates reduces the validity of absolute accuracy measurements. Rank correlation and MAE were therefore used only for comparative analysis within this benchmark (Table 2).

For metalloproteins, MBind showed moderate rank agreement, with a Spearman correlation of ρ = 0.45 and a MAE of 2.15 kcal/mol. GNINA showed similar rank agreement, while GNNSeq and PLAIG showed stronger rank agreement. Boltz-2 showed the lowest numerical deviation among the ML methods, while StructureNet showed inverse rank agreement. Among the non-ML methods, SwissDock and Webina showed the highest rank agreement, with ρ = 0.90 for both methods. Webina also showed the lowest numerical deviation. MolModa showed strong rank agreement and low numerical deviation, while CB-Dock2 and 1-ClickDock showed weaker rank agreement (Table 2). The primary support for the performance of MBind in the metalloprotein set originates from the ligand pose RMSD analyses.

For nonmetalloproteins, MBind showed stronger rank agreement, with ρ = 0.81 and a MAE of 1.62 kcal/mol. Webina showed the same rank correlation and a similar MAE of 1.59 kcal/mol. GNINA also showed strong rank agreement, with ρ = 0.77. Boltz-2 showed moderate rank agreement and the lowest numerical deviation among the ML methods. GNNSeq, PLAIG, and StructureNet showed weak inverse rank agreement. SwissDock also showed inverse rank agreement, while CB-Dock2, 1-ClickDock, and MolModa showed weak positive rank agreement. MBind and Webina showed the highest rank agreement in the nonmetalloprotein set. MBind also showed lower numerical deviation than GNINA, GNNSeq, PLAIG, StructureNet, SwissDock, CB-Dock2, and 1-ClickDock (Table 2).

#### 2.3.3. Per Target Performance Differences

We computed paired differences (MBind RMSD vs. baseline RMSD) for each target and each comparison method (Table S7). Negative values indicate lower RMSD for MBind (better pose accuracy), whereas positive values indicate higher RMSD relative to the baseline method. For metalloproteins, MBind showed consistent improvements relative to most ML and non-ML baselines for the majority of targets. Particularly large advantages were seen for targets where baseline methods struggled (e.g., HDAC2, Human Neutral Endopeptidase). MBind showed mixed performance relative to baselines for nonmetalloproteins, with improvements for some targets (e.g., AR, BRD2) and comparable or slightly reduced performance for others (e.g., PXR, CAR).

#### 2.3.4. MBind Docking Performance Shows Improved Accuracy in Metalloproteins

MBind shows stronger performance than several external docking platforms on zinc metalloproteins, which is consistent with how the workflow treats the zinc active site. MBind uses zinc-specific parameterization during docking and map generation, so predicted poses are produced to account for metal coordination behavior. In contrast, the ML baselines measured here generate poses or affinity estimates using learned representations and scoring. In our benchmarking analysis, these models do not explicitly enforce zinc coordination geometry, such as metal-to-ligand distance or coordination angle constraints during pose ranking. As a result, these methods can place the ligand in the correct pocket while still producing unrealistic local geometry at the zinc center. This is an expected outcome of comparing ML models to metal-parameterized methods and validates AutoDock4Zn’s capabilities within the MBind workflow.

AlphaFold 3 also performed well on several metalloprotein targets. Its workflow differs from MBind as it is trained on large datasets of experimentally confirmed protein-ligand complexes rather than relying on zinc ion parameterization. This difference may explain why AlphaFold 3 and other co-folding models can identify the correct binding region but still show some variation in local zinc ligand distance or coordination angle.

**Figure 11:**
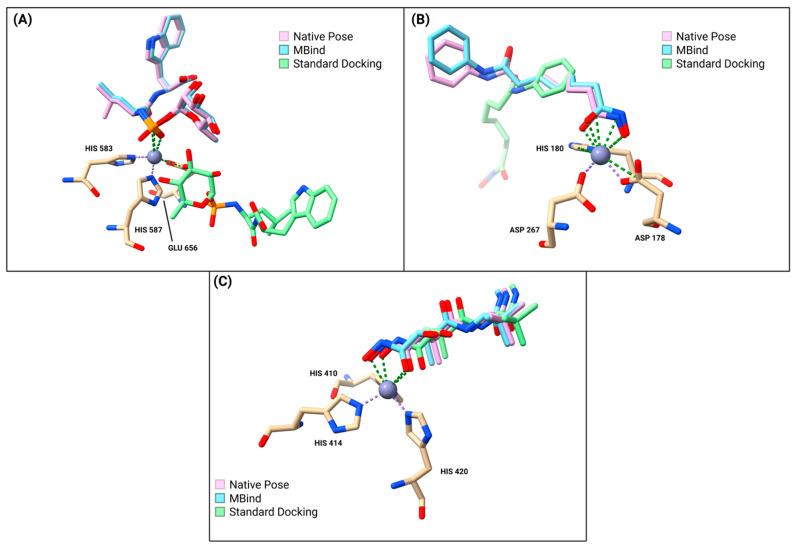
Zinc coordination geometry comparison for predicted docking poses of (**A**) Human Neutral Endopeptidase (1DMT), (**B**) HDAC8 with SAHA, and (**C**) ADAMTS-5. (**A**) Standard Vina docking (without AD4Zn maps) reports Zn-O interatomic distances of 5.6 Å and 7.3 Å. MBind reports interatomic distances of 1.8 Å and 3.1 Å. The native pose has interatomic distances of 1.9 Å and 3.1 Å. (**B**) Standard Vina docking (without AD4Zn maps) reports Zn-O interatomic distances of 11.9 Å and 14.5 Å. MBind reports interatomic distances of 2.1 Å and 2.3 Å. The native pose has interatomic distances of 2.0 Å and 2.0 Å. (**C**) Zn-O interatomic distances for Standard Docking, MBind, and the Native Pose were 2.2 Å and 2.7 Å, 2.1 Å and 2.6 Å, and 2.1 Å and 2.3 Å, respectively. MBind also showed a lower RMSD from the native pose (0.426 Å) compared to the standard docking method (0.782 Å).

Zinc coordination geometry was compared between predicted poses generated by nonmetalloprotein docking and zinc metalloprotein docking. Here, the nonmetalloprotein docking baseline corresponds to AutoDock Vina executed without AD4Zn metal parameterization. Three representative metalloprotein complexes are shown, including Human Neutral Endopeptidase, HDAC8 with SAHA, and ADAMTS-5. The native co-crystallized ligand pose is overlaid with the predicted ligand pose from MBind and the predicted ligand pose from standard AutoDock Vina docking without AD4Zn (Figure 11A,C). The zinc ion and coordinating residues are displayed to highlight local metal site geometry. Zinc-to-ligand coordination distances and key coordination angles were measured from the native pose and from each predicted pose, using the rank 1 pose selected by each method’s default scoring.

For the metalloprotein subset, MBind more consistently reproduces zinc coordination geometry similar to that found in native structures. In contrast, the baseline method shows larger deviations, even when the ligand is placed in the correct binding pocket. In Human Neutral Endopeptidase, MBind reproduces native like Zn-O coordination distances, whereas standard Vina places the ligand oxygens farther from the zinc center (Figure 11A). A similar pattern is observed for HDAC8 with SAHA, where standard Vina substantially overestimates Zn-O distances, while MBind remains close to the native coordination geometry (Figure 11B). For ADAMTS-5, both methods place the ligand closer to the zinc site than in the other examples, but MBind still gives slightly closer Zn-O distances and a lower RMSD than nonmetalloprotein docking (Figure 11C).

Coordination geometry was further evaluated across all eight zinc metalloprotein complexes using the rank 1 MBind pose. For each system, the same zinc atom, protein donor atoms, and ligand oxygen donor atoms were used for the native and predicted poses. Among the benchmarks, the mean absolute Zn to O distance error was 0.21 Å, the mean ligand donor bite angle error was 6.33°, and the mean absolute error for the protein donor to Zn to ligand donor angles was 5.21° (Table S8). Human Neutral Endopeptidase showed the closest recovery of the native geometry. Larger angular deviations were observed for the HDAC8 complexes. These results show that the zinc workflow generally reproduced the native metal-ligand distances and individual coordination angles, although the overall surrounding coordination geometry did not always match the native structure. Individual Zn-to-ligand donor distances and matched coordination angles are reported in the Supplementary Information (Tables S9 and S10).

This direct geometric comparison shows that incorporating metalloprotein-specific zinc parameterization is important for preserving coordination geometry at the metal center. Conventional non-ML docking tools such as SwissDock, CB-Dock2, Webina, 1-ClickDock, and MolModa show comparable BFE estimates for some complexes but higher variability on metalloproteins. This likely shows the difficulty these methods have in reproducing metal site geometry for such targets. The improved metalloprotein pose accuracy and more stable affinity trends observed for MBind are consistent with its zinc workflow.

#### 2.3.5. Cross-Docking Analysis

**Figure 12:**
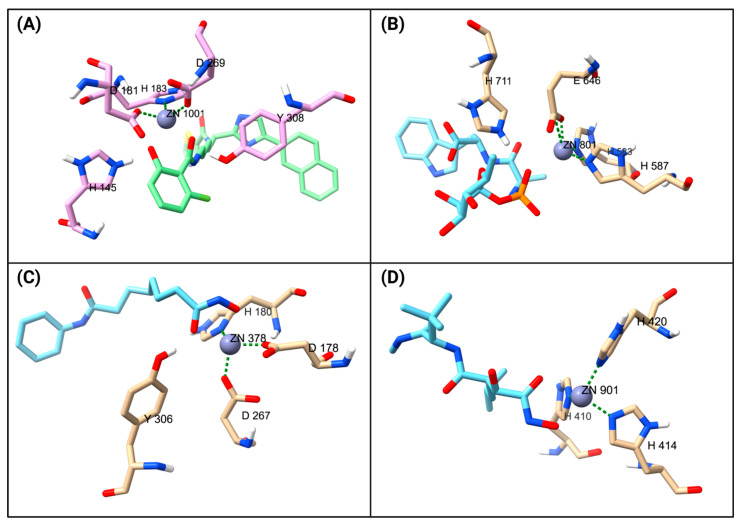
Representative cross-docking pose overlaps generated using MBind. Predicted ligand poses were compared with the corresponding aligned experimental ligand conformations for the best-performing receptor structure of each protein: (**A**) HDAC2 shown in purple with WBA shown in green (PDB ID: 6G30, RMSD = 0.255 Å), (**B**) human neutral endopeptidase shown in tan with Sampatrilat shown in blue (PDB ID: 6XVP, RMSD = 1.261 Å), (**C**) HDAC8 shown in tan with SAHA shown in blue (PDB ID: 1T69, RMSD = 1.556 Å), and (**D**) ADAMTS-5 shown in tan with LA3 shown in blue (PDB ID: 3LJT, RMSD = 1.604 Å).

MBind was further evaluated through a cross-docking analysis using three alternative receptor structures for HDAC2, human neutral endopeptidase, HDAC8, and ADAMTS-5 [40,41,42,43,44,45,46,47,48,49,50]. Overall, 8 of 12 cases produced RMSD values below 2.0 Å, corresponding to a success rate of 66.7%. The mean RMSD was 3.056 Å, while the median RMSD was 1.598 Å. HDAC2 showed the most consistent performance, with all three poses below 0.5 Å. Human neutral endopeptidase and HDAC8 each produced two successful cases, while ADAMTS-5 produced one. Representative overlaps are shown in Figure 12, and the raw cross-docking results are provided in Table S11. Due to the small sample size, these results are intended to provide an example of MBind performance under cross-docking conditions rather than comprehensive validation. The higher RMSD values observed in some cases also show that performance remains sensitive to differences in receptor conformation.

#### 2.3.6. Protonation-State Sensitivity Analysis

Across the three proteins, pH effects were seen. Leukotriene retained the same binding geometry, although its docking score became gradually less favorable from pH 6 to 8 (Table S12). This pattern shows that protonation changes may affect local electrostatic or hydrogen bond interactions without disrupting the overall pose. ADAMTS-5 showed even less sensitivity. Both the RMSD and docking score remained nearly unchanged (Table S12), which indicates that docking repeatedly favored the same non-native orientation across all three conditions. In contrast, HDAC2 showed a strong response at pH 8. The large increase in RMSD was accompanied by a substantial loss in docking score (Table S12). This shift may reflect a protonation change near the zinc center that weakened metal interactions with the ligand. Together, these results show that protonation changes may affect docking score or disrupt both pose and binding strength when pH-dependent active site interactions are affected. The raw data for the pH sensitivity analysis are provided in Table S12.

## 3. Rigor and Reproducibility

Users can download the GitHub repository, which includes the MBind code and benchmark input data folder, and launch MBind using the Streamlit interface to reproduce the results (GitHub at: https://github.com/sivaGU/MBind, accessed on 23 April 2026. The web-based version of MBind is available at: https://mbindgui.streamlit.app/, accessed on 23 April 2026). By using the supplied benchmark receptor and ligand PDBQT files, users can execute docking runs with the same study settings and workflow order used for the manuscript analyses. After docking is complete, users can compare the generated docking outputs with the reported pose RMSD and ΔG summary results described in the manuscript. The repository includes the MBind code, benchmark inputs, and configuration settings needed to rerun the workflow end-to-end, consistent with the procedures referenced in the Data Availability section. To reproduce the results, the user will have to (1) download the MBind repository and included benchmark data folder, (2) launch the GUI locally or run the execution script, (3) load the provided receptor and ligand PDBQT files and execute docking using the specified settings, and (4) analyze the resulting docking poses and binding energies using the procedures described in the manuscript. All benchmarking was done locally using an NVIDIA GeForce RTX 4090 GPU with 16,384 CUDA cores, 24 GB GDDR6X memory (384-bit), and a 2.52 GHz boost clock. All reported results from MBind were created using a fixed MBind workflow and can be reproduced directly from the materials provided in the MBind GitHub repository, which can be accessed at https://github.com/sivaGU/MBind. In addition, the repository provides a requirements.txt file to assist in software reproducibility. The repository also provides a commit hash corresponding to the exact version used in this manuscript. Where random seeds can be fixed, seeds are fixed. Otherwise, repeated runs were performed and summarized. Together, the repository, benchmark inputs, evaluation scripts, and MBind GUI interface provide all materials necessary to fully reproduce the workflow and results presented in this study.

## 4. Strengths

A key strength of this study is the development of MBind. MBind supports zinc, iron, copper, and magnesium metalloprotein and AutoDock Vina docking within one interface. It also automates complex technical and computational steps that may limit metalloprotein docking accuracy. The steps automated include metal map creation, grid map generation, job retry controls, and docking execution. Moreover, the platform is implemented as a web-based GUI, which removes the need for command line usage. MBind was validated through the redocking of native, co-crystal ligands into their respective zinc metalloproteins and nonmetalloproteins. This produced pose overlaps within accepted RMSD ranges (≤2.0 Å, relative to native conformations), which supports the ability of the workflow to reproduce native poses under co-crystal-defined redocking conditions. MBind also performed better than many pose-generating ML models, cofolding models, and non-ML docking tools for zinc metalloproteins. MBind also produced low RMSD values for nonmetalloprotein complex prediction as well. In addition, MBind incorporates the GNINA ML model into the same interface, which allows users to run ML-based docking without requiring command line usage. Another strength of MBind is its ability to handle multiple ligands efficiently. Batch docking and automated output generation make it easy for users to go from input preparation to pose evaluation and score comparison.

## 5. Limitations

The primary limitation of the current version of MBind is that the docking was mainly designed for zinc metalloproteins. The benchmark dataset used in this study is relatively small and may not capture the full diversity of metalloprotein binding sites. The benchmark is also largely focused on zinc-containing complexes, which may bias performance toward zinc-optimized workflows. Additionally, the reported accuracy may be inflated due to redocking rather than docking where the binding location is unknown. Another limitation is that the benchmarking pool mixes methods that do not all solve the same prediction task; therefore, some comparisons are informative but not fully direct. Although MBind has now been extended to support additional divalent metal ions, including Mg^2+^, Fe^2+^, and Cu^2+^, these implementations are preliminary and have not yet undergone extensive benchmarking between diverse test sets. The current results for these metals therefore show workflow compatibility rather than fully validated performance. MBind previously relied primarily on AD4Zn parameterization for zinc within the AD4 workflow, and newly introduced metal-specific parameter files for Mg^2+^, Fe^2+^, and Cu^2+^ require further validation for coordination environments and ligand types. Metalloproteins with more flexible active sites may require further parameters of the platform, as MBind docking uses rigid receptors and depends on the input protonation and tautomer states of ligands. Rigid receptor docking can overestimate performance for flexible binding sites near metal centers, since conformational changes are not shown and the crystal geometry may favor redocking accuracy. Binding free energy comparisons were performed using IC_50_-derived ΔG values, which provide an approximate measure and may not fully reflect true thermodynamic binding affinities. Docking results may also be sensitive to receptor preparation choices, including protonation states, atom typing, and metal parameterization, which can strongly affect outcomes in metalloprotein complexes. Therefore, all predictions should be measured with experimental validation when possible. Despite these limitations, MBind provides a large improvement in accessibility and workflow integration relative to existing metal docking tools.

## 6. Future Directions

MBind predictions should be paired with further analysis and experimental data. Such data would allow direct comparison with MBind’s scoring and binding orientations. Future MBind software will focus on integrating other divalent metal ions such as manganese and calcium. Calcium-specific parameterization would account for its higher coordination numbers and flexible, irregular geometries. Future validation may prioritize calcium-dependent phospholipase A2 enzymes and calpains. Computational validation would examine pose RMSD, calcium-ligand distances, coordination numbers, and coordination angles. Experimental validation may include enzyme activity and inhibition assays to compare predicted binding with measured ligand effects. Additionally, MBind should expand into a machine learning-based prediction platform by incorporating trained ML models. ML-based scoring may initially be incorporated as a post-docking rescoring step, with deeper integration into pose generation explored after further validation. Future studies should also add scoring uncertainty estimates and QM/MM visualization modules to help in understanding the binding results. Additionally, MBind should incorporate molecular dynamics (MD) simulations or interfaces to external MD pipelines. A local MD link that exports prepared receptor complexes to MD workflows allows users to analyze metal centers, solvation effects, and dynamic ligand interactions. This process would not be possible through rigid docking alone. Altogether, these improvements can help expand MBind’s capabilities for a more comprehensive metal binding analysis.

## 7. Materials and Methods

### 7.1. Software Architecture and Tool Implementation

**Figure 13:**
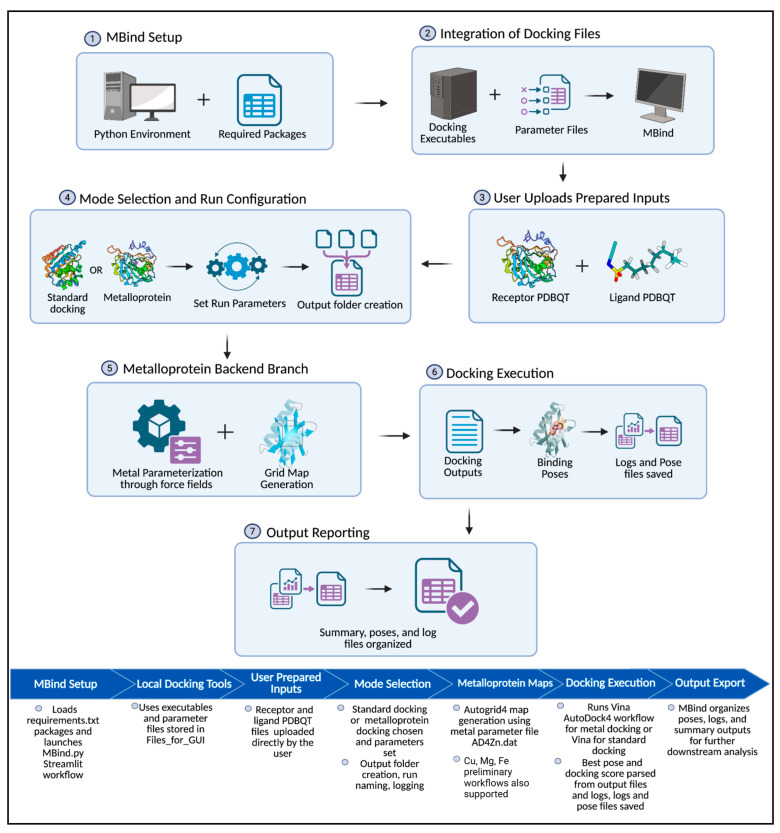
MBind Workflow. The workflow begins with MBind setup in Python and loading required packages (Steps 1 and 2). Users then upload prepared receptor and ligand PDBQT files (Step 3). Based on the selected workflow, MBind prepares docking parameters. For zinc metalloproteins, MBind applies AutoDock4Zn parameterization using AD4Zn.dat, and a similar strategy for Mg, Cu, and Fe ions, to generate AutoGrid maps (Steps 4 and 5). Docking is then executed using AD4 or AutoDock Vina (Step 6). Finally, MBind saves and organizes run outputs for download, which include log files and poses (Step 7).

**Figure 14:**
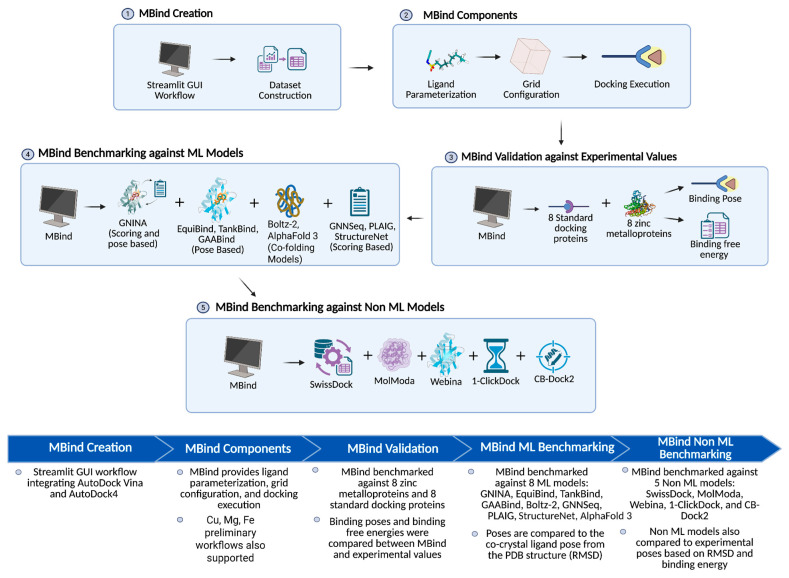
MBind Front End Workflow. The workflow begins with the upload of ligand and receptor files through the Streamlit GUI, where the front-end pipeline constructs the docking dataset (Step 1). MBind then performs ligand parameterization, grid configuration, and docking execution for receptor complexes (Step 2). MBind was benchmarked on a set of 8 zinc metalloproteins and 8 non-metalloprotein docking targets, with predicted binding poses and free energies compared against experimental reference values (Step 3). MBind was then benchmarked against nine ML-based comparison methods grouped by output type: pose-generating methods (GNINA, EquiBind, TankBind, and GAABind), cofolding models (Boltz-2 and AlphaFold 3), and affinity prediction tools (GNNSeq, PLAIG, and StructureNet) for comparative performance evaluation (Step 4). MBind was compared against five non-ML docking platforms (SwissDock, MolModa, Webina, 1-ClickDock, and CB-Dock2) to measure performance relative to conventional docking tools (Step 5).

MBind was developed as a unified platform for nonmetalloprotein and metalloprotein docking. The tool is implemented in Python (v3.12.3) with a Streamlit front end (v1.37.0) and a Python backend (Figure 13). Core dependencies include AutoDockTools (v1.5.7), AutoDock Vina (v1.2.5), AD4 (v4.2.6), and AutoGrid4 (v4.2.6). MBind requires all receptor and ligand files to be in the .PDBQT format, as the platform does not perform internal ligand or receptor file format conversion. The backend configures docking parameters, executes docking through subprocess calls, and stores outputs in structured folders. Additionally, the full GUI version contains approximately 3400 lines of code implemented in Python 3.12.3 (GitHub at: https://github.com/sivaGU/MBind, accessed on 23 April 2026. The web-based version of MBind is available at: https://mbindgui.streamlit.app/, accessed on 23 April 2026). Executables were prepared for both local and web usage. The overall workflow for the front end is described below (Figure 14).

The interface combines AutoDock Vina and AD4 to enable users to execute metalloprotein and nonmetalloprotein docking directly through the interface. The docking software is executed through Python’s subprocess calls, which allows for real-time status tracking. The GUI includes sections for receptor and ligand upload, gridbox definition, grid map commands, and docking status. Additionally, docking on Streamlit Cloud has CPU and memory limits and is best for small runs, while large-scale docking should be done through local execution. The platform also incorporates a user-configurable automated docking retry function, with a user-defined timeout threshold set through the GUI. The timeout is defined as the maximum allowed runtime for a docking process. A retry is triggered only when the docking process exceeds this user-specified time limit. The retry setup does not inspect intermediate logs, error codes, or partial result files. Upon timeout, MBind initiates docking using an exhaustiveness multiplier between retries. The maximum number of retry attempts is also defined by the user at runtime. If the docking process exceeds the timeout threshold, the job is terminated.

The program also automatically includes all atom types needed for zinc metalloprotein docking. The metalloprotein workflow supports zinc ions using AutoDock4Zn parameterization via the AD4Zn.dat file. Zinc metal coordination was modeled using force field-based AutoDock4Zn parameters, and no coordination geometry constraints were used. This AutoDock4Zn parameterization is specific to zinc and represents the primary validated metalloprotein workflow in MBind. Preliminary parameter files and workflows were developed for copper, magnesium, and iron using a similar parameterization strategy. However, these ions do not support the use of AutoDock4Zn and are not yet validated to the same extent as the zinc workflow.

Once map parameters are selected, AD4 is run directly in the working directory. During this step, all produced grid maps and grid parameter files are stored automatically. For output handling, MBind renames and organizes all PDBQT files, log files, and score summaries into structured files. The platform generates CSV files containing docking scores for Vina and AD4 workflows. The platform can be used either locally or through Streamlit Cloud. This makes the platform accessible for different operating systems. A limited “MBind Demo” mode is provided using prepackaged input files hosted on GitHub for users to test metalloprotein docking functionality. MBind is freely available for download with complete user documentation at https://github.com/sivaGU/MBind, and the web-based GUI is available at https://mbindgui.streamlit.app/.

### 7.2. Receptor and Ligand Preparation

All receptor complexes were obtained from the RCSB Protein Data Bank (PDB) [51]. The receptor and its co-crystallized ligand in each structure were separated. The ligand’s original coordinates were retained as the reference pose for docking evaluation. Receptors were prepared in AutoDockTools (MGLTools, AutoDock 4.2) by removing crystallographic waters, hydrogen addition, and assigning Kollman charges [22]. These receptors were then exported into PDBQT format [22]. Structures were manually inspected to confirm that no alternate locations were present. Cofactors, buffer molecules such as glycerol, sulfate, and phosphate, and metal ions were retained only when part of the crystallographically resolved ligand binding environment. They were also retained if they were expected to affect the active site. Molecules were removed if they were distant from the co-crystallized ligand binding region and did not directly interact with either the ligand or the catalytic metal center in the deposited structure. For metalloproteins, the metal ion and its immediate coordinating environment were retained. This preparation was applied to a benchmark set composed of eight metalloprotein and eight nonmetalloprotein targets [43,46,52,53,54,55,56,57,58,59,60,61,62,63,64,65]. The corresponding PDB identifiers are provided and summarized in the Supplementary Materials (Table S1). Docking was performed with rigid receptors that include a Zn active site through AD4 map generation. Protonation states were assigned using the default AutoDockTools protonation tool at approximately neutral pH. Manual adjustments were not needed after inspection of the protonation states. Therefore, Zn-coordinating residues and ligand donor atoms were not reprotonated during docking. This approach is a simplification, as metalloprotein docking is sensitive to local protonation and coordination. Using default protonation without manual adjustment may affect docking accuracy for some targets, which is a limitation of this workflow.

Ligands were prepared through MGLTools and used in PDBQT format. Energy minimization of receptors and ligands was skipped to preserve the co-crystallized binding sites, as these geometries have been experimentally validated. Additionally, grid box center coordinates and dimensions were defined per target and were reused for methods that require an explicit search space (Table S1). Grid boxes were centered on the co-crystallized ligand binding site without automated pocket detection. All top-ranked docked complexes (rank 1) and benchmarking outputs are available in the project GitHub repository for reproducibility (https://github.com/sivaGU/MBind, accessed on 29 July 2026).

### 7.3. Benchmarking of MBind Against ML-Based Pose, Cofolding, and Affinity Prediction Methods

ML-based docking performance was evaluated using TankBind, GNINA, GAABind, GNNSeq, StructureNet, PLAIG, EquiBind, AlphaFold 3, and Boltz-2 [25,26,27,28,29,30,31,32,33]. To keep benchmarking consistent, all methods were run on the same panel of metalloprotein and nonmetalloprotein targets. Furthermore, these were run using a fixed set of ligand inputs. The ML models include pose prediction tools (GNINA, EquiBind, TankBind, GAABind), affinity prediction tools (StructureNet, GNNSeq, PLAIG), and cofolding approaches (AlphaFold 3 and Boltz-2), which differ in their inputs and outputs. AlphaFold 3 predicts complex structures but does not provide binding free energy, IC_50_, Kd, or other affinity values. Therefore, it was included only in the RMSD pose analysis and excluded from BFE comparisons. For each ML method, a separate benchmarking protocol was defined. These protocols included input formatting, preprocessing assumptions, site definition, pose generation, and pose selection criteria (Table S2). All ML docking models were measured using rigid protein inputs with no external structural relaxation applied, with the exception of cofolding models such as Boltz-2 and AlphaFold 3. Methods that required a defined search region or binding pocket had sites specified using the same grid box dimensions reported in the Supplementary Materials (Table S1). Newer cofolding models such as ESMFold2 can accurately predict protein structure, but they do not include metal atom potentials or the local interaction environment needed for accurate metalloprotein docking [66]. Thus, ESMFold2 was not included in ML benchmarks.

Ligand input and parameters were explicitly defined for the pose-generating ML docking models. GNINA prepares receptor-ligand structures in the supported 3D formats and returns a ranked list of poses. EquiBind, TankBind, and GAABind were run in their standard modes by using the required protein structure input and ligand format required by each model. For ML models that accept SMILES input, ligand generation and internal preprocessing were handled by each model’s default pipeline. Any required pocket specification was constrained to the same co-crystal-defined binding region. Boltz-2 and AlphaFold were run using their standard inputs and produced ranked complex outputs, with AlphaFold 3 predictions generated using the Protenix server under server default settings (Table S2) [67]. For all pose-only methods, if a tool produced multiple poses, the measured pose was the highest-ranked pose.

GNNSeq was run as a sequence-based affinity predictor using protein sequence features and ligand representations. PLAIG was run using its required ligand and protein format. Models that perform affinity prediction without pose generation (StructureNet, GNNSeq, and PLAIG) were excluded from RMSD-based pose accuracy analysis as detailed in the Supplementary Materials (Table S2). Pose accuracy for pose-generating methods was measured using DockRMSD without the use of hydrogens. Symmetry-aware atom mapping was used to compute the minimum RMSD relative to the native pose [39]. Experimental IC_50_ values were converted to BFE under standard conditions by using IC_50_ as an approximation of the equilibrium constant. The exact ΔG conversion equation, thermodynamic assumptions, and temperature are defined in Equation (1). The raw benchmarking results for ML models are reported in the Supplementary Materials (Table S3a,b).

### 7.4. Benchmarking of MBind Against Non-ML Docking Tools

The performance of non-ML docking was measured using Webina (v1.0.5), SwissDock, CB-Dock2, 1-ClickDock, and MolModa [34,35,36,37,38]. No parameter modification beyond explicit grid box specification was done for any non-ML docking tool. Each docking tool was run on the same metalloprotein and nonmetalloprotein benchmarking set. For Webina, SwissDock, 1-ClickDock, and MolModa, the same grid center coordinates and box dimensions (x, y, z) were used for each receptor as listed in Table S1. These aforementioned tools with user accessible site definition were manually adjusted to match MBind. When exact grid box control was not available, the closest equivalent site definition allowed by the interface was used.

MBind was also benchmarked against CB-Dock2, a cavity detection blind docking platform. CB-Dock2 was run in blind mode, with the top-ranked cavity overlapping the co-crystallized ligand binding site used for analysis. All other predicted cavities from CB-Dock2 were excluded from pose accuracy and affinity comparisons to maintain consistency. This approach allows for a controlled comparison for methods using the same binding site, but it does not reflect how CB-Dock2 is typically used in fully blind docking and may affect direct comparison. While blind docking typically searches the entire protein surface, we restricted the analysis to the cavity corresponding to the experimental binding site. Pose accuracy was measured using the DockRMSD tool [39]. Additionally, experimental IC_50_ values were used to approximate BFE using Equation (1). For MBind benchmarking, AutoDock Vina was run with exhaustiveness = 128 and num_modes = 10. Default scoring settings were used with a fixed random seed to incorporate exact reruns. All MBind docking workflows, such as AutoDock Vina, AD4, and AutoGrid4, were executed locally using an NVIDIA GeForce RTX 4090 GPU with 16,384 CUDA cores, 24 GB GDDR6X memory (384-bit), and a 2.52 GHz boost clock due to Streamlit Cloud CPU and memory limits. Raw benchmarking data for non-ML models are reported in the Supplementary Materials (Table S3c,d). Comparisons were separated into direct and exploratory categories. Direct comparisons used the same receptor-ligand pairs, binding site information, rank 1 pose selection, and RMSD method wherever supported. Blind docking, cofolding, and affinity-only methods were treated as exploratory because equivalent input information and outputs could not be provided.

### 7.5. Generation of Mg^2+^, Cu^2+^, and Fe^2+^ Specific AD4 Force Field Files

Magnesium-specific AD4 parameter files were generated by using the standard AutoDock4 parameter set and adapting the file structure of the AD4Zn workflow for Mg^2^. The standard AD4_parameters.dat file served as the base. A magnesium-specific file (AD4Mg.dat) was created in the same format as the zinc parameter file. Magnesium atom types were already present in the base AD4 parameter set. Therefore, no additional elemental atom types were introduced. The standard AD4 parameter block was retained, and a tetrahedral magnesium pseudoatom (TM) replaced the zinc-specific pseudoatom used in the original AD4Zn workflow [20]. Mg^2+^ commonly forms octahedral coordination in proteins. However, a tetrahedral pseudoatom was used to match the AD4Zn workflow and to define the docking metal site directly. Therefore, this is a practical approximation rather than an exact match to magnesium coordination.

The Mg atom entries were taken directly from the default AutoDock4.2.6 parameter set and retained without modification [68]. Mg was assigned R_ii_ = 1.30, ε_ii_ = 0.875, *vol* = 1.5600, and *solpar* = −0.00110 [68]. Within the AutoDock4 convention, R_ii_ represents the sum of the van der Waals radii of two identical atom types. The ε_ii_, *vol*, and *solpar* values follow the same format used for standard AD4 metal definitions [22]. The TM pseudoatom was defined as a zero-interaction virtual site. It follows the same noninteracting format used in AD4Zn. This allows it to act as a geometric placeholder for tetrahedral coordination without introducing van der Waals or solvation terms [20]. A merged parameter file (AD4_MgTM.dat) was created by appending the AD4Mg.dat contents to AD4_parameters.dat using the same method implemented in MBind. The final file was checked for correct formatting, proper parameter block structure, and correct placement of the TM pseudoatom. This produced an exploratory magnesium-compatible force field for metalloprotein docking.

Iron and copper AD4 parameter files were generated using the same approach. An Fe-specific file was created by using the standard AutoDock4 Fe atom definition and the AD4Zn file structure. The zinc pseudoatom was replaced with a tetrahedral iron pseudoatom (TF). TF was defined with zero interaction terms and therefore functions only as a geometric placeholder rather than a calibrated iron-ligand interaction potential. The standard AD4 parameter block and file structure were retained. Iron atom types were already present in the base AD4 parameter set, so no new elemental atom types were required. The Fe atom parameters were taken directly from the default AutoDock4.2.6 parameter set and retained without modification. Fe was assigned R_ii_ = 1.30, ε_ii_ = 0.010, vol = 1.8400, and solpar = −0.00110 [68].

A Cu specific file was generated in the same format. Cu atom definitions were added because copper is not included in the default AD4 parameter set. Cu was assigned R_ii_ = 1.40, ε_ii_ = 0.550, vol = 1.7600, and solpar = −0.00110. These values were introduced as an exploratory user-defined atom definition to allow AutoGrid4 map generation, and were not independently calibrated as a general Cu^2+^ force field. A tetrahedral copper pseudoatom (TQ) was introduced to maintain consistency with the MBind metalloprotein workflow, and therefore functions only as a geometric placeholder. The same tetrahedral setup was used for Fe and Cu to keep the workflow consistent for metals, even though these metals can have different coordination shapes in real complexes. Thus, the TF and TQ pseudoatoms represent practical geometric approximations rather than validated descriptions of Fe or Cu coordination. Merged parameter files for Fe and Cu were created using the same append-based procedure described above. These files allow for AutoGrid4 map generation and docking of Fe- and Cu-containing complexes using metal force fields.

### 7.6. Docking of Magnesium, Iron, and Copper Metalloprotein Complexes

Using the magnesium-specific parameterization described above, six magnesium-containing receptor-ligand complexes (7XGI, 2F8Z, 3S68, 4ZQF, 5CG5, and 8FNG) were docked using the same MBind workflow applied throughout the manuscript. All receptor structures were obtained from the RCSB Protein Data Bank [69,70,71,72,73,74]. For each complex, the receptor and co-crystallized ligand were separated, and the ligand coordinates were retained as the reference pose for docking measures. Receptors were prepared in AutoDockTools by removing crystallographic waters, adding hydrogens, assigning Kollman charges, and exporting the structures in PDBQT format. Ligands were prepared using the same workflow, and no additional energy minimization was performed.

Grid box center coordinates and dimensions were defined around the co-crystallized ligand to ensure docking within the active site. During AutoGrid4 map generation and docking, the merged magnesium parameter file (AD4_MgTM.dat) was included so that Mg^2+^ active sites were treated using the standard AutoDock4 Mg atom parameters and the exploratory TM pseudoatom definition. Fe and Cu containing complexes were docked using their corresponding merged parameter files. The Fe file contained the standard AutoDock4 Fe atom parameters, whereas the Cu file contained the exploratory user-defined Cu atom parameters described above. These files contained TF and TQ pseudoatom definitions, respectively, to allow consistent treatment within the same AD4-based metalloprotein workflow. Docking was performed with rigid receptor structures and metal-aware grid maps. This procedure allows all six magnesium complexes to be docked within the same MBind workflow. It also includes four Fe- and Cu-containing complexes while applying exploratory AD4-compatible parameter files for metalloprotein active site representation.

### 7.7. Zinc Coordination Geometry Analysis

Zinc coordination geometry was evaluated for the eight zinc metalloprotein complexes in the primary benchmark. The native crystallographic ligand pose and the rank 1 MBind pose were analyzed for each system. The rank 1 pose was selected using the original docking output order before the coordination geometry was examined. A fixed atom assignment was defined for each complex. The same zinc atom, protein donor atoms, and ligand oxygen donor atoms were used for the native and predicted poses. Protein donor atoms were identified from the zinc coordination records in the original PDB structure. Ligand donor atoms were assigned according to the coordinating functional group in the crystallographic ligand.

Zinc to ligand donor distances were calculated for each selected ligand oxygen atom. The ligand donor bite angle was defined by the two ligand donor atoms and the zinc center. Protein donor to zinc to ligand donor angles were calculated for each matched combination of a crystallographic protein donor atom and ligand donor atom. For every measurement, the absolute error was calculated relative to the corresponding native value. Summary values included the mean and maximum absolute zinc to ligand donor distance errors, the ligand donor bite angle error, and the mean and maximum absolute protein donor to zinc to ligand donor angle errors. The same procedure was used for the native and MBind poses in all eight complexes. For the three representative systems shown in Figure 11, zinc to ligand donor distances were also calculated for the rank 1 standard AutoDock Vina pose generated without AutoDock4Zn parameterization. Summary values for the eight zinc complexes are reported in Table S8, individual native and MBind distances are reported in Table S9, and matched angles are reported in Table S10.

### 7.8. Cross-Docking Analysis

A cross-docking analysis was performed for HDAC2, human neutral endopeptidase, HDAC8 with SAHA, and ADAMTS-5. Three alternative receptor structures were selected for each protein. The original benchmark ligand was docked into each alternative rigid receptor structure. The same MBind workflow and docking settings were used for all cases. Each receptor was aligned with its reference structure. The grid box was centered on the aligned experimental binding site. Rank-1 poses were compared with the aligned experimental pose using DockRMSD [39]. Cross-docking was considered successful when RMSD was below 2.0 Å. The complete results are provided in Supplementary Table S11.

### 7.9. Protonation-State Sensitivity Analysis

Protonation-state sensitivity was evaluated for leukotriene A4 hydrolase, ADAMTS-5, and HDAC2. Receptors were prepared at pH 6, 7, and 8 using PDB2PQR v3.7.1 and PROPKA v3.5.1 [75]. Zinc ions were restored at their crystallographic coordinates. The structures were converted into rigid PDBQT files using Open Babel. The assigned hydrogen atoms were retained. The same ligand, grid box, and docking settings were used for each pH condition. Ligand protonation was kept constant. Rank-1 RMSD values and docking scores were compared across the three pH conditions. The complete raw data are provided in Supplementary Table S12.

## 8. Conclusions

This study presents MBind, a web-based workflow that combines AutoDock4, AutoDock Vina, and optional GNINA support for zinc metalloprotein and nonmetalloprotein docking. The primary validated metalloprotein workflow uses AutoDock4Zn-style zinc parameterization with automated grid map generation to reduce manual setup requirements, thereby improving reproducibility. In redocking benchmarks, MBind produced low RMSD values for zinc metalloprotein targets and closely reproduced native zinc coordination geometries with better consistency than nonmetalloprotein docking. Binding energy trends were comparable to those from the evaluated ML and non-ML methods, while the performance on nonmetalloproteins was similar to established docking tools. Although preliminary workflows for magnesium, iron, and copper are included, these require additional validation. Future work should expand benchmarking to use larger datasets, additional metal ions such as manganese and calcium, and experimental validation. ML-based pose prediction and scoring approaches should also be incorporated in future improvements. Overall, benchmarking of MBind shows that integrating docking methods within a single workflow improves consistency in metalloprotein docking, while maintaining comparable performance among nonmetalloprotein targets.

## Figures and Tables

**Table 1:** Benchmark receptor set used in this study. PDB identifiers for the eight zinc metalloproteins and eight nonmetalloprotein targets used for docking validation and benchmarking. Each structure contains a co-crystallized ligand that serves as the reference pose for redocking accuracy and BFE comparison.

Receptor	PDB ID	Grid Box Parameters (x, y, z)	Center Coordinates (x, y, z)
ADAMTS-5	3HY7	22, 28, 26	15.045, 4.229, −6.35
Human Neutral Endopeptidase	1DMT	26, 30, 30	27.882, 42.765, 34.683
HDAC 2	7KBG	25, 25, 25	47.447, 9.539, −37.297
HDAC 8 with Hydroxamic Acid	5FCW	24, 22, 24	36.385, 12.919, 122.956
HDAC 8 with SAHA	1T69	22, 24, 24	30.243, −3.216, −15.22
hACE	1O86	30, 28, 30	40.553, 32.798, 47.285
LTA4H	1GW6	30, 28, 26	33.767, 5.025, 1.384
HDAC10	7U6B	34, 30, 28	−28.115, −40.887, 34.611
Hsp90 with geldanamycin	1YET	24, 22, 20	40.899, −46.138, 65.505
Hsp90 with PU-H64 inhibitor	2FWY	24, 24, 20	2.266, 10.022, 25.383
AR	2PIV	30, 30, 30	27.457, 2.568, 5.486
BACE1	3EXO	26, 30, 28	34.522, 44.099, −1.621
MDM2	4OCC	22, 20, 20	−21.466, −10.622, −35.273
PXR	5X0R	32, 28, 28	32.327, 14.28, 24.871
BRD2	6E6J	22, 24, 20	19.079, −10.235, −7.098
CAR	1XNX	30, 26, 28	21.321, 28.883, 82.198

**Table 2:** Rank agreement and numerical deviation between predicted model scores and fixed IC_50_-derived activity estimates. Spearman rank correlation was used to measure agreement in score ordering. Mean absolute error, median absolute error, and root mean squared error were used to describe numerical deviation. Reported IC_50_ ranges were represented by their geometric midpoint, and the same fixed reference value was used for all methods.

Protein Class	Method Category	Method	Spearman Rho	MAE	Median Absolute Error	RMSE	n
Metalloprotein	MBind	MBind	0.45	2.15	1.35	2.78	8
Metalloprotein	ML-associated	GNINA	0.46	2.54	1.98	3.25	8
Metalloprotein	ML-associated	GNNSeq	0.85	1.53	1.23	1.9	8
Metalloprotein	ML-associated	PLAIG	0.88	2.78	2.41	3.09	8
Metalloprotein	ML-associated	StructureNet	−0.24	1.81	1.83	2.08	8
Metalloprotein	ML-associated	Boltz-2	0.64	1.25	0.58	1.83	8
Metalloprotein	Non-ML	SwissDock	0.9	1.29	1.37	1.49	8
Metalloprotein	Non-ML	CB-Dock2	0.55	2.07	2.29	2.34	8
Metalloprotein	Non-ML	Webina	0.9	1.11	0.98	1.31	8
Metalloprotein	Non-ML	1-ClickDock	0.36	1.38	0.9	1.85	8
Metalloprotein	Non-ML	MolModa	0.79	1.19	0.63	1.6	8
Nonmetalloprotein	MBind	MBind	0.81	1.62	1.41	1.83	8
Nonmetalloprotein	ML-associated	GNINA	0.77	1.67	1.53	1.88	8
Nonmetalloprotein	ML-associated	GNNSeq	−0.07	1.92	1.83	2.31	8
Nonmetalloprotein	ML-associated	PLAIG	−0.07	1.88	1.97	2.25	8
Nonmetalloprotein	ML-associated	StructureNet	−0.07	1.88	1.87	2.26	8
Nonmetalloprotein	ML-associated	Boltz-2	0.55	1.44	1.2	1.94	8
Nonmetalloprotein	Non-ML	SwissDock	−0.45	2.97	2.86	3.6	8
Nonmetalloprotein	Non-ML	CB-Dock2	0.06	1.68	1.93	1.82	8
Nonmetalloprotein	Non-ML	Webina	0.81	1.58	1.28	1.8	8
Nonmetalloprotein	Non-ML	1-ClickDock	0.1	2.08	1.9	2.53	8
Nonmetalloprotein	Non-ML	MolModa	0.17	1.58	1.57	1.84	8

## Data Availability

All the data generated in this study are presented in the main manuscript, and additional data are available in the Supplementary Materials and on GitHub. The MBind source code, benchmark input files, configuration settings, MBind-generated outputs, and evaluation scripts are publicly available to allow full reproduction of MBind results. Raw outputs from third-party docking tools are not distributed, but can be regenerated using the provided inputs and documented workflows. MBind is available on GitHub at: https://github.com/sivaGU/MBind, accessed on 23 April 2026. The web-based version of MBind is available at: https://mbindgui.streamlit.app/, accessed on 23 April 2026.

## Supplementary Materials

The following supporting information can be downloaded at: https://www.mdpi.com/article/10.3390/molecules31152703/s1, Table S1: The grid box parameters and center coordinates prepared within AutoDockTools for each receptor docking procedure. All docking dimensions were targeted at the active site of each receptor; Table S2: Docking tool category, version/commit, protein and ligand input types, site definition method and details, samples/poses generated, inference settings, pose selected for RMSD, protein rigidity/flexibility, hardware, and notes; Table S3: (a) Docking Scores and RMSD Values of Ligands Bound to the Active Site of Metalloproteins used for MBind Validation in ML models, (b) Docking scores and RMSD values of ligands bound to the active site of standard (nonmetalloprotein) proteins evaluated using ML docking models for MBind benchmarking, (c) Docking scores and RMSD values of ligands bound to the active site of zinc metalloproteins evaluated using non-ML docking tools for Mbind benchmarking, and (d) Docking scores and RMSD values of ligands bound to the active site of standard (nonmetalloprotein) proteins evaluated using non-ML docking tools for Mbind benchmarking; Table S4: Bootstrap resampling results summarizing RMSD and binding free energy error across metalloprotein and non-metalloprotein benchmark sets for ML and non-ML docking methods; Table S5: Mean absolute error, Spearman correlation coefficient, and Spearman p-value for each method according to protein class and tool class; Table S6: Calibration coefficients, training and test sample sizes, test mean absolute error, and test Spearman correlation coefficient for each method according to protein class and tool class; Table S7: Target-specific RMSD values for MBind and comparator docking methods and the corresponding RMSD differences between MBind and each method; Table S8: Docking scores, zinc–ligand distances, distance errors, bite angles, protein–ligand angle errors, and local head-group angle errors for native and MBind poses; Table S9: Zinc–ligand distance measurements and corresponding native values, signed errors, and absolute errors for native and MBind poses; Table S10: Ligand bite-angle, protein–ligand coordination-angle, and local head-group-angle measurements and corresponding native values, signed errors, and absolute errors for native and MBind poses; Table S11: Cross-docked receptor PDB IDs, RMSD values, mean and median RMSD values, successful cases below 2.0 Å, and success rates; Table S12: Rank-1 RMSD values and docking scores obtained at pH 6, 7, and 8.
